# Escalate protein plates from legumes for sustainable human nutrition

**DOI:** 10.3389/fnut.2022.977986

**Published:** 2022-11-04

**Authors:** Nisha Singh, Priyanka Jain, Megha Ujinwal, Sapna Langyan

**Affiliations:** ^1^Department of Bioinformatics, Gujarat Biotechnology University, Gandhinagar, Gujarat, India; ^2^National Institute of Plant Genome Research, New Delhi, India; ^3^Amity Institute of Molecular Medicine and Stem Cell Research (AIMMSCR), Amity University, Noida, Uttar Pradesh, India; ^4^ICAR-National Bureau of Plant Genetic Resources, New Delhi, India

**Keywords:** amino acid, balanced diet, legumes, nutrition security, physico chemical properties, plant protein

## Abstract

Protein is one of the most important, foremost, and versatile nutrients in food. The quantity and quality of protein are determinants of its nutritional values. Therefore, adequate consumption of high-quality protein is essential for optimal growth, development, and health of humans. Based on short-term nitrogen balance studies, the Recommended Dietary Allowance of protein for the healthy adult with minimal physical activity is 0.8 g protein/kg body weight (BW) per day. Proteins are present in good quantities in not only animals but also in plants, especially in legumes. With the growing demand for protein, interest in plant proteins is also rising due to their comparative low cost as well as the increase in consumers’ demand originating from health and environmental concerns. Legumes are nutrient-dense foods, comprising components identified as “antinutritional factors” that can reduce the bioavailability of macro and micronutrients. Other than nutritive value, the physiochemical and behavioral properties of proteins during processing plays a significant role in determining the end quality of food. The term “complete protein” refers to when all nine essential amino acids are present in the correct proportion in our bodies. To have a balanced diet, the right percentage of protein is required for our body. The consumption of these high protein-containing foods will lead to protein sustainability and eradicate malnutrition. Here, we shed light on major opportunities to strengthen the contribution of diversity in legume crops products to sustainable diets. This review will boost awareness and knowledge on underutilized proteinous foods into national nutritional security programs.

## Introduction

The world’s population is expected to reach 10 billion people by 2050. Already, one-third of the current population is affected by malnutrition and undernutrition, which are mostly caused by a lack of healthy foods and a shift in lifestyle (1). There is a need for research into healthy and diversified crops that can grow in harsh or substandard conditions (2, 3). Only three crops provide half of the world’s calories (wheat, rice, and corn), however these grains are low in protein and several minerals (4). Legumes supplement cereals in the diet; they provide significant protein, necessary amino acids and minerals, and can be used in place of meat when it is unavailable or pricey. The Leguminosae belongs to the Fabaceae family (beans or legumes) and is the third biggest and most economically important flowering plant family, with 650 genera and almost 20,000 species of herbs, shrubs, trees, and climbers (5). The legume subfamily includes the Papilionoideae, Caesalpinoideae, Mimosoideae, and Swartzioideae. As an essential component of the human diet, people have been growing legumes as crops for millennia. Alfalfa, clover, lupines, green beans and peas, peanuts, soybeans, dry beans, broad beans, dry peas, chickpeas, mung beans, lentils, and moth beans are all important parts of the human diet, especially in underdeveloped nations (6). Therefore, it is important to add these nutritional diets to people’s daily routine.

Legumes are pod/fruit containing dry grains and have the tendency of nitrogen fixation because the roots of leguminous plants contain rhizobium bacteria which take the nitrates and convert them into nitrogen. This helps to make protein-rich food and, therefore, leguminous plants are considered as a source of protein (7). Since human civilization developed, pulses and cereals were linked, and proteins in both pulses and cereals were able to meet the essential amino-acid requirements of humans and animals (8). Legumes provide approximately one-third dietary protein nitrogen for (9) and average legume consumption is 21 g/person/day, worldwide (10). Pulses belong to the Legume family and, hence, every pulse is a legume, but this is not true vice versa; for example, dry beans, dry peas, chickpeas, lentils, etc. are pulses while legumes are peanuts, soybeans, fresh peas, etc. (11). Pulses have been consumed for many years as part of traditional diets around the world. Approximately 1,000 legumes have been identified but only 20 of them are widely cultivated. These legumes are all grown extensively across Asia and the Pacific. Protein-rich legumes are also cholesterol-free, high in dietary fiber, and low in saturated fat. Legume seeds crude protein ranges from 15.5 to 42% (Table 1). India accounts for 26% of global yield and is the largest producer as well as consumer of pulses (12, 13). The degree of inclusion vs. avoidance of animal-based foods varies widely among human dietary habits around the world (14, 15). The argument that specific amino acids are “missing” from certain plant meals are patently untrue. All 20 amino acids, including the nine essential amino acids, are found in plant diets (16).

**TABLE 1:** Protein content, PD, PER, CS, BV, NPU, micronutrients and amino acids information of cultivated legumes.

Sr. No.	Name	Scientific name	Protein content (g/100 g)	PD (%)	PER	CS	BV (%)	NPU (%)	Micronutrients value	Amino acids	References
1.	Field pea	*Pisum sativum* L.	26	80	2.4	NA	75	60	Zinc-34 mg, Copper-6.3 mg, Manganese-15.6 mg, Iron-58.1 mg, Nickel-3.4mg	Rich in all essential amino acids except methionine, lysine, and threonine	(24, 158, 159)
2.	Soybeans	*Glycine max*	13	80	1.7	NA	74	61.5	Iron-3.21 mg, Molybdenum-0.11 mg, Copper-0.23 mg, Phosphorous-19 mg, Sodium-2.35 mg	Rich in all essential amino acids	(31, 32, 85, 160)
3.	Mung bean	*Vigna radiata* L.	23	70.2	4.29	76.2	64	56.3	Iron-4.4 mg, Magnesium-166 mg, Sodium-13.2 mg, Calcium-114 mg Zinc-2.1 mg, Potassium-414 mg	Rich in leucine, phenylalanine, valine, lysine, and histidine	(37, 40, 95, 161)
4.	Cowpea	*Vigna unguiculata*	24	55.49	2.65	50.88	NA	NA	Zinc-1.29 mg, Iron-2.51 mg, Potassium-278 mg, Calcium-24 mg, Magnesium-53 mg, Sodium-4 mg, Phosphorous-156 mg	Rich in leucine, lysine and phenylalanine; limited in methionine and cysteine	(50, 53, 162, 163)
5.	Common bean	*Phaseolus vulgaris*	24	67⋅47	2.95	39.07	NA	NA	Potassium-355 mg, Phosphorous-40 mg, Zinc-1.12 mg, Iron-2.1 mg, Magnesium-70 mg, Copper-0.209 mg	Higher in methionine compared to most legumes except soybean	(58, 164)
6.	Pigeon pea	*Cajanus cajan* L.	22	59.2	1.87	65	68.5	40.6	Sodium–32.5 mg, Magnesium–138.8 mg, Iron—51.5 mg, Calcium–581 mg	Limited in isoleucine, lysine, methionine, and tryptophan	(67, 68, 165, 166)
7.	Chickpea	*Cicer arietinum* L.	21	83.8	2.32	NA	67.51	58.63	Calcium- 49 mg, Magnesium-48 mg, Potassium-291 mg, Phosphorus-168 mg, Iron-2.89 mg, Zinc-1.53 mg, manganese-1.03 mg	Limited in tryptophan, methionine, and cysteine	(75, 167)

*PD, Protein Digestibility; PER, Protein efficiency ratio; CS, Protein chemical score; BV, Biological value; NPU, net protein utilization; NA, Not available.

Legumes contribute to agriculture and the environment in addition to being a key source of food and nourishment for the population. Their nitrogen-fixing ability, for example, can improve soil fertility and assist in avoiding soil erosion as a crop cover. Some legumes are supposed to prevent or treat hypertension, diabetes, and cancer; therefore, they have medical benefits as well (17). As a result, their effects on essential dietary components such as amino acids, lipids, carbs, vitamins, and minerals are given specific attention. Furthermore, in recent years, more individuals have substituted vegetable protein for animal protein, increasing demand for legumes, which are the primary source of plant proteins. To meet this demand, researchers must focus on nutritional profiling of various legumes, increase the use of underutilized legumes, develop low-cost, innovative value-added legume products, educate consumers about the nutritional value of legumes, and find new ways to encourage the use of existing legumes (10). The current review paper also focuses on the proven and potential health advantages connected with legume consumption.

## Important legume food crops with nutritional composition and their applications

**FIGURE 1:**
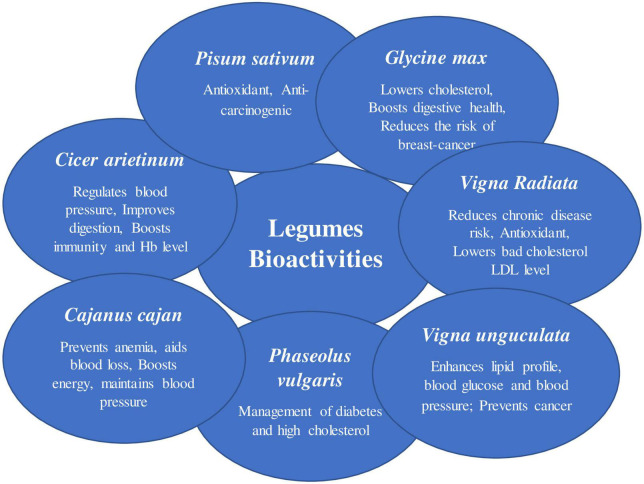
Depicting multi-uses of legume crops.

**FIGURE 2:**
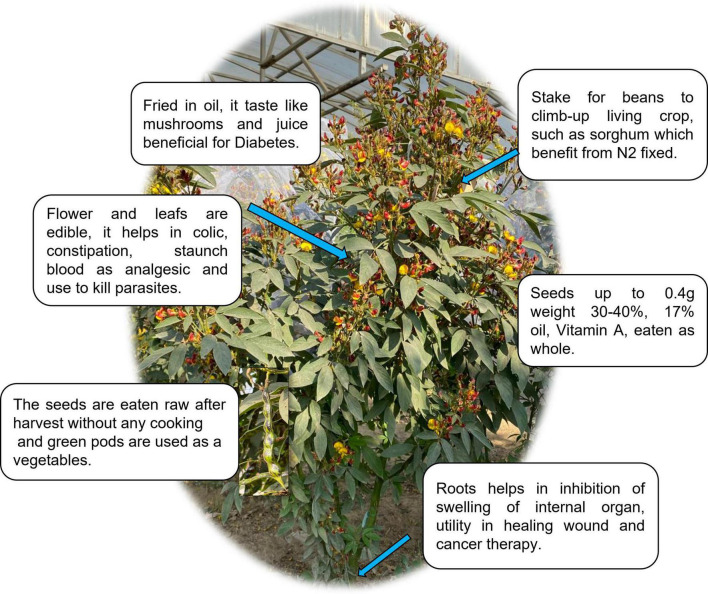
Different bioactivities of the legumes. A summary of the legume plant (pigeonpea): A multifunctional crop containing roots, seeds, and leaves that can be used in a variety of ways.

Legumes have been grown in both the “Old and New Worlds” for thousands of years. It’s probable that their long history as food crops is due to their easy-to-harvest seeds, which have a low water content and can be stored for long periods of time when dry. These characteristics, together with their high protein content and simplicity of cultivation, make legumes attractive crops (Figure 1) (6). Legumes have a range of bioactivities, ultimately leading to various health-promoting benefits (Figure 2).

### Pea (*Pisum sativum*)

The pea is the only species in the Leguminosae family, under the genus Pisum. Pea is one of the world’s most important legumes, with global annual production estimated at roughly 13.5 million metric tons and a producer price of over 200 USD/ton, and at present it is grown in over 90 countries (18). In peas, protein content largely varies based on genetic and environmental factors (Table 1). Peas also contain high quantities of lysine, which might help to compensate for lysine shortage in cereal-based diets (19). The nutritional values are defined by amino acid content. Peas have a good content of valine, arginine, and methionine, although a poor content of cysteine and glutamic acid. In raw pea, the protease inhibitors present are responsible for decreasing *in vitro* digestion (20). Because of their high carbohydrate, protein, and other nutrient content, peas have long been an important part of the human diet. The content and characteristics of starch, protein, fiber, vitamins, minerals, and phytochemicals in peas are primarily responsible for their health advantages (21). Fiber from the seed coat and cotyledon cell walls improves gastrointestinal function. Pea starch decreases the glycemic index and lowers starch digestibility due to its intermediate amylose level (22). Peas include several phytochemicals that were previously thought to be antinutritive. Polyphenolics, found in colored seed coat types, have antioxidant and anticarcinogenic properties, as well as saponins, which have hypocholesterolemia and anticarcinogenic properties, and galactose oligosaccharides, which have beneficial prebiotic effects in the large intestine (23, 24). Other than protein, peas also contain minerals like zinc (27.4–34 mg kg^–1^), copper (5.2–6.3 mg kg^–1^), manganese (9.0–15.6 mg kg^–1^), nickel (2.3–3.4 mg kg^–1^), and iron (47.7–58.1 mg kg^–1^), while potassium and magnesium are somewhat lower (25). Peas are a good source of vitamin C, E, and other antioxidants; these help to improve the immune system, reduce inflammation, and lower the risk of chronic condition like diabetes, heart disease, and arthritis (26).

### Soyabean (*Glycine max*)

The soybean (*Glycine max*) is one of the world’s most important crops. It is known as the “world’s miracle crop” because of its nutrient-dense content, which makes it ideal for animal feed and human diets. In addition to serving as an oil seed crop and cattle feed, the crop is a good source of protein for human diets and as a biofuel feedstock (27). Soybean is a type of oil seed that contains a variety of nutrients such as protein, carbohydrates, vitamins, and minerals. Soybeans are composed of 36% protein, 19% oil, 35% carbohydrate (17% dietary fiber), 5% minerals, and a variety of other nutrients, including vitamins (28). Mineral content in soybean is in the higher range (0.2–2.1%) for chloride, sodium, potassium, calcium, and phosphorus, while in the lower range (0.01–140 ppm) for chromium, magnesium, copper, iron, arsenic, silicon, cobalt, lead, sulfur, and zinc (29). Soybean contains all essential amino acids required for the balanced functioning of the human body like lysine, isoleucine, leucine, phenylalanine, tyrosine, threonine, methionine, cysteine, tryptophan, histidine, and valine (Table 1). It is a good crop for preventing protein deficiency and serving as a bridge for vitamin “A” absorption (30). Soybeans have many health benefits: it keeps you full for a long time, lowers cholesterol level, boosts digestive health, effects on insulin secretion and energy metabolism, reduces the risk of breast cancer, and is compatible with many specialized diets (31–33).

### Mung bean (*Vigna radiata*)

Mung bean (*Vigna radiata* L.) is a popular pulse eaten around the world, particularly in Asian countries, and has a long history of use in traditional medicine. It has long been recognized as a high-quality source of protein, dietary fiber, minerals, vitamins, and considerable levels of bioactive substances such as polyphenols, polysaccharides, and peptides, making it a preferred functional food for maintaining good health (34). The essential amino acids like leucine (1.847%), phenylalanine (1.443%), valine (1.237%), tryptophan (0.26%), isoleucine (1.008%), arginine (1.672%), lysine (1.664%), methionine (0.286%), threonine (0.782%), and histidine (0.695%) are highly present in mung bean (35, 36). The proteins present in mung bean (60%) have higher *in vitro* digestibility compared to soybean protein (65%) (37). Apart from protein content, mung bean also serves as a good source of minerals that are found in different ranges like magnesium (129–166 mg/100 g), sodium (8.7–13.2 mg/100 g), iron (3.4–4.4 mg/100 g), calcium (81–114 mg/100 g), zinc (1.2–2.1 mg/100 g), and potassium (363–414 mg/100 g) (29). Our body requires mineral for various crucial functions, including enzyme reactions, protein synthesis, glucose control, and muscle and nerve function (15, 38). The polyphenols, polysaccharides, and polypeptides found in mung beans all have antioxidant properties, which can help to prevent various diseases (39) by lowering bad LDL cholesterol levels and reducing chronic disease risk (37, 40). The mung bean and its extracts have demonstrated excellent health benefits, including hypoglycemic and hypolipidemic effects, as well as having antihypertensive, anticancer, anti-melanogenesis, and immunomodulatory properties (41).

### Cowpea (*Vigna unguculata* L.)

Cowpeas (*Vigna unguculata* L.) are a member of the Fabaceae family. Cowpeas are grown for both grains and veggies. It is one of the most nutrient-dense African indigenous vegetables with the potential to improve food and nutrition security. The crop originated in West and Central Africa, from where it expanded to Latin America and Southeast Asia through cultivation and production (42). Cowpea has high protein content, and it differs with variety (43). The protein profile of cowpea is complex and unique. Cowpea leaves are high in protein, vitamins like provitamin A, folate, thiamine, riboflavin, and vitamin C, as well as minerals like calcium, phosphorus, and iron (44). The essential amino acids present in cowpea are cysteine, histidine, methionine, leucine, isoleucine, lysine, threonine, and tryptophan. Cowpea proteins are composed of amino acids like phenylalanine, valine, leucine, and lysine, which are found in higher levels than sulfur-containing amino acids. The amino acids are utilized during seed development; because of this, the free amino acid content is higher in immature seeds compared to mature seeds (45, 46). Cowpea leaves have long been used to produce food and feeds, nutraceuticals, micronutrients, and natural antioxidants. (47). Alpha-tocopherols, flavonoids, lycopene, and anticancer agents are among the antioxidants found in the leaves (48). In cowpea (*Accession* OAA96/30), the mean value of the mineral elements calcium, chloride, sodium, magnesium, and potassium is 892.1, 177.7, 106.4, 2477.0, and 14710.0 μ*g/g*, respectively (49). Cowpea seeds have different ranges of micronutrients in different genotypes. The range for Zn is 33.9–69.2 μg.g^–1^, for Fe is 45.1–67.0 μg.g^–1^, 5.2–8.1 μg.g^–1^ for Cu, and 10.1–17.4 μg.g^–1^ for Mn (50). Cowpea possesses iron in high quantities, which eliminates anemia and assists in protein metabolism, which is essential for RBCs and hemoglobin production. Manganese also maintains the structure and strengthening of bones (51). It assists in the formation of bone by regulating the enzymes and hormones which are involved in the process of bone metabolism (50).

Proteins, peptides, and protease inhibitors in cowpea have been shown to improve lipid profile, blood glucose levels, and blood pressure, as well as help to prevent cancer by reducing the growth of various cancer cell lines (50, 52, 53). Cowpea protein isolate can be used in food items as an alternative. Protein isolation is a viable option for reducing antinutritive effects and increasing digestibility and bioavailability of leguminous amino acids. In impoverished nations, a mixed meal of legumes and grains can compensate for inadequacies or low levels of lysine and sulfur amino acids in cereals and grain legumes, respectively (46, 54).

### Common bean (*Phaseolus vulgaris*)

Domestication of the common bean began around 8,000 years ago in central Mexico and South America. These were isolated events that led to the formation of two large genetic pools: Mesoamerican, which stretches from northern Mexico to Colombia, and Andean, which stretches from Peru to Argentina (55). Common beans are an important dietary proteinaceous source to large populations of these semi-dry global regions, contributing to about 15% of calorie intake and up to 36% of total daily protein in parts of Africa and the Americas (56). Seeds of common bean have ∼25% protein along with minerals like Mg, Cu, Ca, Fe, Zn, and vitamins such as folate (“FoodData Cent.,” n.d.). Beans have useful levels of mineral micronutrients like copper (9.1–11.6 mg kg^–1^), selenium (381–500 μg kg^–1)^, potassium (18,854–23,175 mg kg^–1^), magnesium (1,845–2,383 mg kg^–1^), and zinc (24.8–33.3 mg kg^–1^) (26). Common beans have 25–30% of the recommended content levels of iron in food and are known as rich sources of several other minerals (57). Common bean has a lesser genetic complexity than soybean, and the new genetic information available for the Andean and Mesoamerican pools makes it an appealing model for studying molecular mechanisms like symbiosis and nutritional deficiency adaptation (56). Common beans, without a doubt, have a lot of potential as a nutraceutical food. Encouragement of its consumption could aid in the prevention of chronic diseases, diabetes, and high cholesterol, which are on the rise around the world (58). The processing of ordinary beans into a range of goods may expand the number of products available for consumption (59).

### Pigeon pea (*Cajanus cajan*)

Pigeon pea is a perennial plant in the Fabaceae family. Pigeon pea is a popular legume crop in South Asia, providing high protein content and nutritional benefits for over a billion people (60). Red gram, congo pea, gungo pea, and no-eye pea are some of the other frequent names for this tropical legume. Due to its higher yield under extreme environmental circumstances, such as heat, drought, and low soil fertility, this hardy legume has a significant potential positive influence on the lives of impoverished farmers when compared to other legumes (61). Pigeon pea contains higher amounts of amino acid like aspartic acid, lysine, leucine, arginine, and glutamic acid (Table 1) (62). The sulfur-containing amino acids (methionine and cystine) are limited in major legumes but are found in pigeon pea (63). It has a good number of health-promoting phytochemicals in addition to protein and fiber. Phenolic acids, flavonoids, tannins, saponins, and phytic acid are the most common phytochemicals identified in pigeon pea seeds. Antioxidant, antidiabetic, and anti-inflammatory properties are the most common bioactivity of these components (64, 65). The functional characteristics of pigeon pea flour make it a good ingredient for culinary products such as bread, pasta, and nutritional bars, and it can be used as a gluten-free cereal substitute (66). Recent data suggests that bioactive compounds found in pigeon peas play an important role in modifying the gut microbiota and, as a result, can lower inflammation. Animal models have also been used to examine the prebiotic potential of non-digestible raffinose family oligosaccharides (67). Pigeon pea helps in maintaining blood pressure, preventing anemia, aiding weight loss, and boosting energy (67, 68).

Pigeon pea can also be used as a substitute for artificial nutrition formulae that cause low-level inflammation. Now it is used as a unique nutritional ingredient in foods such as biscuits, noodles, pasta, and sausages because of its high fiber and protein content, gluten-free status, low glycemic index, antioxidant levels, and functional features such as fat absorption and water-binding capability (69).

### Chickpeas (*Cicer arietinum* L.)

Chickpeas (*Cicer arietinum* L), often known as garbanzo beans or Bengal gram, are the third most significant variety of legume. The chickpea is divided into two types: Kabuli and Desi. Desi grain has 327 kcal/100 g of energy, while Kabuli grain has 365 kcal/100 g (70). Chickpea grains have a greater protein content than any other pulse crop, with 60–65% carbohydrate, 6% fat, and between 12 and 31% protein. Chickpeas are high in protein, carbohydrates, and all the essential amino acids. Chickpea seed has the highest digestibility as compared to other dry edible legume, with 23% protein, 64% total carbohydrates, 47% starch, 5% fat, 6% crude fiber, 6% soluble sugar, and 3% ash. Calcium, magnesium, potassium, phosphorus, iron, zinc, and manganese are all abundant in chickpeas (71). When compared to legumes like the common bean and soybean, chickpea seeds have a similar protein content. Chickpea protein has a high bioavailability and digestibility (48–89.01%) (72). Chickpea is a rich source of several minerals like manganese (21.9–25.4 mg kg^–1^), copper (6.6–8.7 mg kg^–1^), iron (48.6–55.6 mg kg^–1^), (73) zinc (21.1–28.3 mg kg^–1^), selenium (629–864 μg kg^–1^), and magnesium (1,525–1,902 mg kg^–1^) (26). These micronutrients are responsible for a variety of health benefits like improving digestion, aiding weight management, and reducing the risk of heart disease. Chickpeas have higher levels of selenium (731 μg kg^–1^) but lower levels of calcium compared to other legumes (26). Chickpeas can boost a food’s nutritional value while also lowering its acrylamide concentration. Acrylamide is a toxic chemical that can be found in meals including bread, crackers, and chips. The application of chickpea flour and protein could be a novel technique to lower the amount of acrylamide in these goods. The sensory and textural qualities are affected by the inclusion of chickpea flour (74). The protein quality of legumes is improved by heat treatment because heat inactivates several heat liable anti-nutritional factors Chickpeas have several possible health benefits, and when combined with other pulses and grains, they may be able to reduce the risk of cardiovascular disease, type 2 diabetes, digestive illnesses, and several other malignancies. It regulates blood pressure, improves digestion, and boosts immune health and hemoglobin level (75). Overall, chickpeas are a valuable pulse crop with a wide range of nutritional and health benefits (Table 1 and Figure 2) (76).

## Functional properties of legume proteins

Legumes are a low-cost protein source, which makes them valuable and healthy components whether utilized as flours, concentrates, or protein isolates (PIs) (77). The protein content of legumes differs based on the legume species. Seed proteins are divided into three categories: structural, storage, and physiologically active. Enzymes, lectins, and enzyme inhibitors are the most physiologically active proteins (78).

### Legume protein type

The predominant protein components in legume seeds are globulins (35–80%) and albumins (2–37%). The primary globulins include legumin (11S) and vicilin (7S), while albumins include enzymes, enzyme inhibitors, and lectins (79, 80). Albumins are water-soluble proteins that contain enzymes, protease inhibitors, amylase inhibitors, and lectins. Globulins account for nearly 70% of legume seed proteins, are predominantly composed of the 7S, 11S, and 15S proteins, and are defined as protein extractable in dilute salt solutions. Pea protein is mostly made up of the 7S/11S globulin (salt-soluble, 65–80% of total) and albumin 2S (water-soluble, 10–20%) protein classes, whereas soybean has large number of storage proteins like globulin and prolamin. Mung bean is enriched with storage proteins like albumin (25%) and globulin (60%) (81). Cowpea includes albumins, globulins, prolamin, and glutelin as major protein types while common bean is mainly composed of albumin and globulin (47.56 and 44.64%, respectively). Pigeon pea storage protein found within pulses are globulins, albumins, and glutelin. Albumins and globulins are the primary proteins present in chickpeas, with tiny amounts of glutelins and prolamines present. Legumin and vicilin belong to globulins. The primary storage protein, legumin (360 kDa), accounts for 97% of all globulins. A detailed description of all the legume “protein type” and their comparison with the lentil and peanut is presented in Table 2.

**TABLE 2:** Protein composition of different legumes and their comparison with lentil and peanut.

Legumes	Protein content (%)	Albumin (%)	Globulin (%)	Prolamin (%)	Glutelin (%)	References
*Pisum sativum*	26	18	55	4	3	(168, 169)
*Glycine max*	13	8	35	21	5	(72, 170)
*Vigna radiata*	23	25	40	10	10	(95)
*Vigna unguiculata*	24	10.25	10.25	9.27	6.92	(171)
*Phaseolus vulgaris*	24	47.56	44.64	0.98	0.74	(172, 173)
*Cajanus cajan L.*	22	10	29.2	16	3	(174–176)
*Cicer arietinum L.*	21	12	56	2.8	18.1	(86, 175)
*Lens culinaris*	10.5–27.1	26	44	2	20	(177)
*Arachis hypogaea*	47–55	6–9	12–16	9.3	2.5	(178)

### Physico-chemical and functional properties of the legume proteins

Proteins function as both nutritional and structural building blocks (82). Physical and chemical features of legume proteins, including molecular weight (MW), amino acid content and sequence, structure, surface electrostatic charge, and effective hydrophobicity, are all linked to their functioning. Protein functioning can be altered by physical, chemical, enzymatic, or genetic alterations. The isoelectric points (PIs) are increasingly being used in food products due to their functional characteristics (83, 84). Amino acid profile information on all the legumes under study is provided in the Table 1. Essential amino acids are present in many legumes, while non-essential amino acids, like glutamic acid and aspartic acid, are also present, except in chickpeas (24, 53, 85). MW- Leguminous seeds’ primary storage protein is legumin. Legumin has a MW between 300 and 400 kDa and is a hexamer. Peas are the source of lectin or agglutinin known as Pisum sativum (PSA). PSA is a heterodimer made up of four subunits and has a MW of about 53 kDa. Chickpea legumin has a molecular weight (MW) of between 320 and 400 kDa (86). The amino acid sequence yielded a MW of 20.1 kDa in Glycine max. Cajanus cajan has a MW of 14 KDa (87). The molecular mass of Phaseolus vulgaris is 126 kDa. MBL I and MBL II, two significant lectins, make up the VRA (Vigna radiata agglutinin) protein. 132 kDa is the MW of the tetramer MBL I. In Vigna unguiculata, three globulin subunits with MWs of 110, 76, and 41 kD were discovered to be made up of disulfide-linked polypeptides (88).

Isoelectric point- The pH level at which a specific molecule has no net electrical charge is known as the isoelectric point (89). The protein isoelectric point of legume globulins is thought to be about pH 4.5 (90). Major pea proteins are globulins, which are most soluble above and least soluble below the isoelectric point (4.5), respectively (77). The majority of chickpea proteins have an isoelectric point of 4.5, which is the point at which there is no net charge and no repelling interactions between protein molecules (91). Glycine’s isoelectric point is at pH 6.082, and its isoelectric zone ranges from around pH 4.5 to pH 7.5 (92). Cajanus cajan proteins have an isoelectric point of pH 4.5 where there is no net electrical charge on them (93). Cowpea (Vigna unguiculata) has a conventional solubility pattern with an isoelectric point at pH 6 (94) while *Phaseolus vulgaris* has an isoelectric point between pH 5.2 and pH 6.2 and the isoelectric point of *Vigna radiata* is at pH 4.5 (95).

Hydrophobicity-Food proteins’ structural and functional characteristics, such as hydration, gelation, emulsification, foaming, and adhesion, are known to be significantly influenced by hydrophobicity. Hydrophobicity is anticipated to have a significant impact on the physicochemical characteristics, bioavailability, and overall nutritional quality of proteins found in legumes. Comparing the surface characteristics of myoglobin, bovine serum albumin, and lactoglobulin with those of soybean trypsin inhibitor, concanavalin A, and 7S globulin, legume proteins have a higher hydrophobic surface area than myoglobin and serum albumin, according to molecular surface coloring by amino acid hydrophobicity. Numerous hydrophobic regions on the protein surface of lactoglobulin have been linked to the binding of tiny ligands, particularly phenolics (96). Studies on the specific legumes are scarce.

Solubility-In Legume, globulins protein has reduced solubility at pHs close to the protein isoelectric point and have a hydrophobic nature. The solubility of pulse protein is very low (pH 4 and 6) and the solubility is influenced by ionic strength, pH, freezing, temperature, and heating (97). In Soybean, globular protein forms gel (83) when heated and due to this functional group of globular protein get exposed (79). Gelification is affected by the amount of water, concentration of protein, temperature, and pH. The soy PIs change with the alteration of pH and by proteolytic enzymes. Acylation of legume proteins increases the foaming and emulsifying capacity of protein.

Thermal stability- Thermal behavior and thermal stability are the two major properties of the legume’s PIs. Thermal behavior of protein denaturation (Td) in chickpeas with 70% purity and faba beans with 88% purity was recorded as 205 and 183°C, respectively (98). Td values for bean isolates at 55 and 75% purity were 211.5 and 193.8°C and lentil isolates at 45 and 75% purity were 199.5 and 183.4°C, respectively. The thermal stability of legumes rises with purity (99).

Oil Holding Capacity (OHC) and Water Holding Capacity (WHC)- The OHC and WHC of the legumes ranges from 3.5 to 6.8 gm/gm and 1.8 to 6.8 gm/gm, respectively. Legume PIs have good foaming, emulsion, solubility, and emulsifying activity indexes, and these qualities are maintained even at a high pH (2.0 and 10.0) (100).

Encapsulation has made considerable use of legume proteins. Vitamin B9 (folate), tocopherol, ascorbic acid, and phytase are encapsulated independently using legume PIs (e.g., from pea and chickpea), with encapsulation efficiencies ranging from 62 to 100% (101, 102). In simulated gastric and intestinal fluids, phytase encapsulation with pea protein isolate resulted in a low release rate and high bioaccessibility (103). To encapsulate the vegetable oils and flaxseed and soybean oils, respectively, lentil and red kidney bean protein isolate are utilized (104). Apart from these, melting temperatures can vary greatly depending on the particular main amino acid sequence linked to the particular protein type. The pH and salt concentration of the solution, as well as post-translational changes, such as glycosylation, can significantly affect the stability of the protein structure and, consequently, the melting point. Protein shape, charge, and hydrophobicity, as well as the neutrality of dipoles and the hydration of polar groups, all affect their ability to emulsify. The strength of these interactions influences the stability of emulsions (105).

Du et al. (106) studied the bulk densities, water absorption indices, water solubility indices, oil absorption capacities, emulsion activities, and emulsion stabilities in the whole legume floor and found that these properties ranged from 0.543 to 0.816 g/mL, 4.09 to 6.13 g/g, 19.44 g/100 g to 29.14 g/100 g, 0.93 g/g to 1.38 g/g, 61.14 to 92.20%, and 84.15 to 96.90%, respectively. Further, high hydrostatic pressure (HHP), ultrasound, enzymatic hydrolysis, and a combination of these technologies can be used to increase the functioning of legume proteins (107–109). Another way to change the characteristics of legume proteins is to use enzyme hydrolysis. The antioxidant and anti-inflammatory activities of PIs from pigeon pea, lentil, and chickpea were improved by alcalase and bromelain hydrolysis (109). The above functional modifications change the structural, chemical, and physicochemical properties of proteins (84).

## Impacts of processing techniques on physicochemical properties of legume proteins

Raw bean consumption can be time-consuming, difficult, and, in the worst-case scenario, unsuccessful in terms of the gut’s ability to absorb amino acids. Lupin PIs and defatted wheat meet the required amino acid requirements. Pigeon pea and PIs derived from it are also potential sources of sulfur-containing amino acids that are suitable for human consumption. A range of domestic processing procedures are used empirically to facilitate the absorption of legumes in general, but without understanding the impact on protein digestibility *per se*. Many bean cultivars and processing methods have been examined to assess the potential for enhancing nutritional value and protein utilization (110). The recent plant protein revolution has pushed legumes to the center of consumer and food industry attention (111). These goods can also be used to fortify foods, boost their content, and produce healthy products that are reduced in calories, cholesterol, and fat. Food protein content is also increased by various food processing procedures that may alter its chemical and physical qualities (112). Some of these may potentially boost protein functioning. As a result, they can also be used as functional additives to improve physical qualities such as hydration, oil-holding capacity, and viscosity (113). Soaking, thermal processing, and microwave radiation enhances the bioavailability of the legume proteins (114–116). Other high-intensity thermal procedures include pressure cooking and autoclaving. Both rely on exceeding the typical, ambient boiling point of water in a sealed vessel (pressure cooker) or pressure chamber (autoclave) (117). These rely on milling the legumes into flour before treating them. Baking is an old, conventional thermal process that applies heat using dry air at atmospheric pressure. In general, a dough based on bean flour is produced, rested for some time (leavening), and then baked for more than 30 min at temperatures ranging from 180 to 220°C. Extrusion is likewise based on the transformation of bean flour into a food product (118). Impact of different processing techniques on legume proteins is enumerated in the Table 3. These different processing methods that could affect the legume protein different ways are presented as:

**TABLE 3:** Effect of processing techniques on protein in different legumes.

Legumes	Dehulling	Soaking	Cooking	HHP	Fermentation	References
*Pisum sativum*	Total protein content increased by 2.2% after germination via de-husker	NA	IVPD increased by cooking in water bath.	Protein digestibility, oligosaccharides, phytic acid and trypsin inhibitor activity reduced	IVPD increased by 2–3%.	(179)
*Glycine max*	increased the oil holding, foaming and functional properties	Raffinose content reduced to 22% after soaking seed in water for 16 h	Cooking of seeds reduced the tannin content and improved its IVPD	NA	Fermentation with *Lactobacillus bulgaricus*, the IP6 contents in the seed decreased	(180)
*Vigna radiata*	No change in protein content After manually removing post-soaking	Protein content decreased by 0.2% after soaking in distilled water for 12 h at room temperature	NA	Decreased isoleucine, tryptophan, total sulfur amino acid, and lysine	Increases the protein digestibility	(79)
*Vigna unguculata*	Increased the oil holding, foaming and functional properties.	Soaking seeds in alkaline water for 16 h eliminated raffinose and reduced stachyose by more than 80%	Cooking reduces anti-nutritional content and increase IVPD	NA	Fermentation with *Lactobacillus bulgaricus*, the IP6 contents in the seeds decreased	(180, 181)
*Phaseolus Vulgaris*	NA	Decrease in the protein content 0.2% after soaking in distilled water for 16 h at 30°C	NA	Soaked see drained, mix with oil and salt, boil for 3 h would increase protein digestibility	Increased the potential of flours as functional ingredients by increasing polyphenol and their anti-oxidant activity	(182)
*Cajanus cajan*	NA	Soaking for 6 h helped in detoxification of trypsin inhibitor and tannins and protein content increased by 6.34%	Cooking was more efficient in reducing the levels of lectins, oxalates, saponins and tannins	NA	Fermentation improved the protein solubility	(183)
*Cicer arietinum*	Total protein content increased by 5% after germination via de-husker	Soaked seed and mixed with oil and salt, boil for 3 h. increase the protein content up to 4.51%	Cooking reduced the RFO contents, with the elimination of verbascose	NA	Ground mix with inoculum and fermented for 24 h, increased the protein digestibility by 13.4%	(184)

*HHP, high hydrostatic pressure; IVPD, *in vitro* protein digestibility; NA, Not available.

Cooking- Unprocessed bean, pea, and lentil flour have similar amounts of protein in them, ranging from 24.8 to 28.7%. However, when contrasting different subtypes of cooked legumes, the impact on the protein content varied greatly. Phaseolus vulgaris beans that had protein levels of 24.8 and 26.2% before processing were dramatically reduced to 23.0 and 21.3% (117). All cooked legumes showed a considerable rise in *In vitro* Protein Digestibility (IVPD). The IVPD of the raw samples of peas (*Pisum sativum* L.) varied between 79.9 and 83.5%, while that of the processed samples ranged between 85.9 and 86.8%. After 90 min of heating, the unprocessed chickpea’s IVPD—which was 83.61%—rose to 88.52%. The IVPD was shown to depend on the cooking time (118).

Autoclaving- When compared to the raw form, the crude protein content of beans, peas, and lentils was significantly reduced after autoclaving. Comparatively to cooking, autoclaving raw beans has a different impact on their protein content (119). Like this, autoclaving dramatically raised the IVPD for all species of legumes studied, with the exception of faba bean and lentil. When compared to cooking, autoclaving did not clearly improve protein digestibility. The more severe and intense heat treatment provided by autoclaving did not always appear to be beneficial. Additionally, the IVPD of four distinct legumes was considerably decreased when the autoclaving time was increased from 10 to 90 min (117).

Fermentation- Black beans fermented with the fungus showed a protein digestibility of 48.13%, which is comparable to figures for black beans fermented with Bacillus. The fungus’ capacity to lessen tannins contributed to the improvement in protein digestibility (120, 121).

Baking- The steps involved in making dough, including mixing, rising, and baking, compared to cooking, meant the IVPD was decreased in all but the red lentil. The protein content of green split peas was decreased by this processing procedure while the protein content of yellow split peas increased. Protein content of untreated yellow split peas was 179.23.78%, and after extrusion and baking, it increased to 24.56% (115, 116).

High hydrostatic pressure- HHP treatment enhanced the kidney bean, lentil, and protein hydrolysates’ solubility and emulsifying activity index (107). Following HHP treatment, there were noticeable changes in the secondary structure, including a shift of the amide I and amide II, which demonstrated a decrease in the emulsion stability of lentil PI and kidney bean protein hydrolysate. Chickpea PIs were treated with high-intensity ultrasound, which greatly enhanced their solubility, emulsifying, foaming, and heat-induced gel characteristics (107, 108).

Extrusion- Due to the strong shear and compression, extrusion is a thermal process with a high energy efficiency and is likely the most severe thermal treatment technique. However, the method of extrusion enhanced the nutritious value of legumes (122). The IVPD was markedly enhanced by extruding kidney bean, pea seed, common bean, and faba flour. Extruded kidney beans (100–300 g/kg) were added to broiler diets, and this increased apparent ileal digestibility (AID), which was 77.23–79.03% with feed based on raw kidney beans (117).

There are numerous processing processes available to lower the amount of antinutritional components contained in legumes, boost the bioavailability of macro- and micronutrients, and expand the range of pulse-based products available to consumers (123). To achieve the required outcomes, the processing procedures can be adjusted to the purpose and kind of legumes (124).

## Extraction methods used for legume protein type

Proteomics is a cutting-edge approach for analyzing the protein profiling of a tissue in order to interpret the molecular entities that could be changed in order to generate crops that are resistant to stresses. Because of its high protein content (18–30%) in legumes and ease of digestion (68%), it is a key source of protein, especially for a substantial portion of the Indian population who closely follow vegetarian diets (125). Protein extraction technologies can aid in increasing the yield of extracted protein as well as its nutritional and functional qualities. As a result, a suitable protein extraction method should be used (15). Globulins and albumins are the two main proteins found in legumes. These proteins have a minimal solubility at pH values ranging from four to five (isoelectric point). Protein concentrates and isolates with varied purity and functionality can be generated by modifying protein solubility and utilizing filtration procedures that take advantage of their hydrodynamic properties (84).

Various extraction techniques are used for the extraction of different protein types in the legumes. Air classification and pin milling fractionation of legumes into a light or fine fraction (protein concentrate, defined as having protein contents of 60–75%) and a heavy or coarse fraction (starch concentrate, defined as having protein contents of 90–95%) is done. Using this procedure, whole or dehulled legume seeds are pin milled and then fractionated into “protein” and “starch” concentrations using an air classifier (126). However, the quality of the protein fraction obtained through this procedure is low (38–65%), and additional processing is frequently necessary (127). Alkaline extraction and acidic (isoelectric) precipitation method involves the use of distinct protein solubility and precipitation characteristics. The alkaline and acid pH ranges have higher solubility, but the isoelectric point (about pH 4–5) has the lowest solubility (127). The salt extraction process, also known as micellization, is based on the salting-in and salting-out of dietary proteins. After extracting the protein using a suitable salt solution of the necessary ionic strength, the solution is diluted, producing protein precipitation that can then be recovered by centrifugation or filtration, followed by drying (128).

## Legume protein in human nutrition

Legumes have been a staple of many traditional cuisines around the world for thousands of years. Some of the food uses of the different legumes have been presented in Table 4. Per capita legume consumption has been stable over the last three decades, whereas meat consumption has increased, particularly in several LMIC (low- and middle-income countries). Plant-forward diets, which involve a move away from meat and toward alternate protein sources such as legumes, are seen as a way to reduce greenhouse gas emissions, water usage, and deforestation (129, 130). These legumes have a high vitamin and mineral content, particularly potassium and calcium (in the case of lupines and soybeans), magnesium, iron, and zinc, as well as vitamin B1 (thiamine) and folates. Soybean is a good source of both linoleic acid and α-linolenic acid (15). Due to their low-fat content, the remaining legumes do not play a significant role as providers of fatty acids. Legumes have a high fiber content. Disaccharide and oligosaccharide levels are very high. Sulfur-containing amino acids (methionine and cystine) limit amino acids in faba beans and lupines (131–133). Protein digestibility in all legumes is relatively high, ranging from 89 to 96%. As a result, the Protein Digestibility Corrected Amino Acid Score (PDCAAS) values vary from 81 (lupines) to 96 (peas). These high PDCAAS levels are comparable to animal proteins. Peas and beans, and to a lesser extent lupines, are high in lysine, making them an excellent supplement to cereal proteins, which are poor in lysine. Arginine is also abundant in lupine protein (134). In the future, legumes may provide a sustainable solution to the challenge of providing high-quality dietary protein to the world’s growing population, but this may necessitate significant increases in global legume yields and directing the majority of legume production toward human consumption rather than livestock feed (10).

**TABLE 4:** Food uses of different legumes across the world.

Common name	Food uses
Adzuki beans (*Vigna angularis*)	Japanese desserts and confections, soup ingredients for therapeutic purposes
Anasazi beans (*Phaseolus vulgaris*)	Boiled meal, snack, soup
Black gram (*Vigna mungo*)	Dhal, fermented products (idli, dosa, papad)
Black turtle beans (*Phaseolus vulgaris*)	Bean soup popular in latin American cuisine
Black-eyed peas (*Vigna unguiculata*)	Boiled snack/part of meal, fried cake *akara*, steamed pudding *moi moi* in West Africa
Chickpea (*Cicer arietinum*)	Middle Eastern and Mediterranean foods such as falafel and hummus, Boiled/fried/cooked/crushed snacks, dhal, curry, flour used in bread making, fermented food (*dhokla)*
Faba bean (*Vicia faba*)	Whole food
Kidney beans (*Phaseolus vulgaris*)	Ingredient in Mexican chili; most-consumed legume in America
Lentils (*Lens culinaris*)	Soups and stews; most important legume in India
Mung beans/Green gram (*Vigna radiata*)	Bean sprouts, cooked whole or with sugar into a dessert, soup, flour used for baking, transparent noodles, patties, sweets
Peanut/Groundnut (*Arachis hypogaea*)	Peanut butter, peanut bar, flour, roasted/boiled snacks
Peas (*Pisum sativum*)	Soup, dhal
Soybean (*Glycine max*)	Asian dishes (tofu, natto miso), roasted snacks, milk, yoghurt, sprouted beans, curd, yuba, soy sauce, soy paste, TVP
Tamarind (*Tamarindus indica*)	Pulp used for food and beverage preparation, flour used as soup thickener, remedy in diarrhea and dysentery

Compiled from Semba et al. (10) and Kouris-Blazos and Belski (185).

In recent years, there has been increased interest in the bioactive characteristics of legume-derived proteins and peptides. Furthermore, because legume starch has a low glycemic index, it contributes to a gradual release of glucose. Dietary fibers from legumes help to normalize bowel function and gastrointestinal health Given the present demand, it is likely demand for diverse legume ingredients will increase in the future. The better functionality of legume-based products will contribute to these developments since legume ingredients can produce specialty food products in addition to meeting daily nutritional requirements (111). Legumes will continue to play an important part in human nutrition and health as the recognition of their potential for improved crop environmental sustainability grows.

## Therapeutic use and mechanism of the legume proteins

Due to improved nutritional and functional characteristics of legumes, they can be used in food and nutraceutical applications (135) as well as for various health benefits (108). Products generated from the production or processing of plant-based foods are rich in nutrients. Nutrient-rich diets have been linked to a lower risk of cardiovascular disease, diabetes, hypertension, obesity, and gastrointestinal diseases (136). In addition to the nutritional superiority, legumes are high in bioactive phytochemicals, such as phenolic compounds, and have various bioactivities like antiviral, anticarcinogenic, anti-inflammatory, and antimutagenic, as well as lowering blood cholesterol, blood glucose, blood pressure, and body weight (BW), which is beneficial in coronary heart disease (CHD), osteoporosis, and other degenerative diseases (Figure 2). The therapeutic uses/health benefits of different legume proteins have been explored in Table 5.

**TABLE 5:** Health benefits of various legume proteins.

Legume source	Involved metabolism	Type of study	Beneficial effect	References
Dry beans, peas and peanuts	Cardiovascular and colorectal cancer	Epidemiologic Follow-up study	22% reduction in coronary heart disease (CHD) and 11% reduction in cardiovascular disease (CVD)	(185)
Legumes	Cardiovascular	Meta-analysis	10% decreased risk of CVD and CHD	(186)
Cowpea	Hypocholesterolemic	Endogenous biosynthesis of cholesterol	Lowers blood cholesterol, blood glucose, blood pressure, and body weight	(52)
Cooked beans, chickpeas or lentils	Cardiovascular and diabetes	randomized controlled trial	Reduced hemoglobinA1c (HbA1c) level and reduction in coronary heart disease (CHD) risk	(187)
Five cups/week yellow peas, chickpeas, navy beans, and lentils)	Metabolic syndrome	Randomized controlled trial	Reduced risk factors of the metabolic syndrome	(188)
Beans with low-GI diet	Glycaemia and obesity	randomized, crossover study	Improvement in metabolic control in type-2 obese diabetic patients in weight loss	(188)
Legume	coronary disease	Nutritional intervention and/or dietary manipulation	Prevent the generation of superoxide radical and boost the immune system	(189)
Legume seed extracts	Colon cancer	Test-tube lab research	Inhibition of MMP-9 activity and cell migration in colon carcinoma cells	(190)
Protein isolates of germinated soybean	Cervical cancer	Animal research studies	Decreased in the expression of PTTG1 and TOP2A (therapeutic targets) causing apoptosis of cancer cells	(191)
Common bean	Hypolipidemic properties	Bio-active constituents’ study	Phytosterols, dietary fibers, and resistant starch.	(192)

The quantitative and qualitative protein composition is one of the fundamental aspects in selecting plants for nutritional value, and because globulins are the predominant proteins in legumes, there have been an abundance of studies on their characterization in various species (137). In chickpea, during the germination stage, the increase in proteolytic activity was followed by a modest decrease in trypsin inhibitor activity, which is connected to albumin fraction. Protein properties and mobilization during seed germination have been widely examined, revealing gradual hydrolysis with an unknown mechanism of degradation regulation (138). Many plant species contain basic 7S globulin (Bg7S)-like proteins from soybean seeds. Bg7S was previously assumed to be one of the key seed storage proteins, but later investigations revealed that it was not a storage protein, but rather a protein with multiple activities such as stress response, antimicrobial activity, hormone receptor-like activity, and so on. Bg7S and leginsulin, on the other hand, were discovered to have functional effects on humans, rats, and mice, such as blood glucose management, blood pressure control, and plasma cholesterol control, as well as cancer cell antiproliferative properties (139). It also suggested that seed storage protein consumption may affect adipocyte differentiation *in vivo*, although this mechanism’s effect adipocyte differentiation is not fully understood (72). Peas and lentils include a variety of potentially bioactive components, including protease inhibitors and lectins, which can affect human metabolism in either a favorable or negative way. Some bioactive substances, on the other hand, may be helpful to health. The nutritious quality of the seeds is considerably improved by thermal treatment. Raw and treated seeds are both hypo-cholesterolemic (140). Peas are hypo-cholesterolemic in rats, pigs, and humans. The processes are unknown, but they may be related to fiber and saponins in the seeds (141). On the other hand, Lectins can activate multiple pathways that restrict cancer cell development, with the apoptosis pathway being more effective in specific cell lines by increasing the synthesis of caspases or other proteins involved in the mechanism. Specific genes in this pathway can be down-regulated or up-regulated, resulting in apoptotic suppression or activation, respectively (142). Chickpea lectin inhibited cell growth in human oral cancer cells (KB cells) at a concentration of 37.5 g/mL (IC50) but had no harmful effect on normal human peripheral blood mononuclear cells at a concentration of 600 g/mL (143). Based on the findings and information available, it is possible to conclude that legume protein type (globulins, albumin, prolamin, and glutelin) are effective antibacterial and bioactive agents that can be successfully and efficiently used to understand the therapeutic effects and nutritional benefits of plant legumes, particularly because they are safe natural products that can be prepared at a low-cost, in addition to their well-known rich nutritional value as legume proteins.

## Underutilized and neglected legumes

Obtaining food resources for the world’s growing population is getting increasingly difficult. In this situation, each country must make the most of its existing food supplies (144). However, in most African and Asian nations, where underutilized crops could have addressed malnutrition, hunger, and rural poverty, several legume crops that were formerly essential sources of proteins, edible lipids, fiber, minerals, and other nutritional components are on the verge of extinction (29). In reality, some of these crops—namely marama bean, bambara groundnut, yambean, and lablab—offer enormous promise in terms of climate adaptation and nutritional content. Because of their nutritional makeup, these crops have a lot of potential as future food or feed sources (145). However, these potentially essential crops are in danger of becoming uncommon due to a lack of agronomic, genetic, and food research (146).

The African yam bean (*Sphenostylis stenocarpa*) (AYB) is a popular tropical African food crop with medicinal and pesticidal properties. Although lower than soybean (38%), AYB has around 29 and 19% crude protein in its grain and tuber, respectively (147). Bambara groundnut (*Vigna subterranea*) is not widely consumed in many parts of Africa, although its nutritional and health benefits are well known. In recent years, there has been increasing interest in cultivating V. *subterranea* in arid savannah zones to reduce stress and boost protein availability to the local population (148). The winged bean (*Psophocarpus tetragonolobus*) is a multipurpose legume plant with edible and medicinal portions (149). Vitamins, minerals, protein, and secondary metabolites including phenolic, and flavonoids are abundant in all parts of the plant. Furthermore, winged bean is very resistant to biotic and abiotic stressors, allowing it to thrive in a variety of environments (150).

With help in breeding, transgenic techniques, and agronomic practices, nutritional value of the crops can be enhanced by using biofortification methods (3, 151). In comparison to rice, wheat, and maize, limited analysis has been conducted on biofortification of pulses and legumes in the last decade (29). Biofortification, on the other hand, is one of the more cost-effective ways to address the malnutrition problem among underprivileged and impoverished rural populations (152–154).

These neglected crops are becoming increasingly scarce around the world, and their genetic resources are fast disappearing in all of their natural habitats. As a result, a paradigm shift from the current state of neglect to sustainable cultivation, exploitation, and consumption of the species is required (155). The genetic resources accessible among and within neglected crops have not been fully explored to enhance sustainable use for future food and nutritional security, as well as biodiversity preservation to mitigate the detrimental effects of climate change and abiotic stress. Similarly, a species’ ability to tolerate abiotic stress in the face of biological limiting conditions is critical to its continuing use and survival (156, 157).

## Conclusion

For a healthier lifestyle, legumes are an inexpensive and long-term source of protein, micronutrients, complex carbohydrates, and essential phytochemicals. Their composition is suitable for celiac and diabetes patients as well as consumers concerned with satiety. The variety and versatility of legumes means that they can be added to a wide range of dishes, including soups, stews, curries, and salads (70). Legumes are great crops for reaching the Sustainable Development Goals due to their nutritional, health, and environmental benefits. Therefore, the genetic resources of legumes should be explored using characterization, evaluation, and documentation. This will provide the identification of trait-specific germplasm for further utilization of the same in the national food basket. Legumes impart various health-promoting bioactivities like reduced risk of CHD, lowered cholesterol, and maintained blood sugar level, blood pressure, and metabolic syndrome. There are only a few reports on the mechanism of action, so it would be important to conduct more studies to verify biological activities, not only *in vivo* models but also *in situ* or *ex vivo*, performed not only in the whole seed but with each of its bioactive compounds, to reveal the benefits of its consumption and importance as a functional food. Various plant components, such as carotenoids and flavonoids, have been shown to have beneficial health advantages. Furthermore, research on plant proteins and bioactive compounds is needed for sustainable human nutrition. More research is needed to determine the best ways to introduce plant proteins safely and effectively into the diet because the incorporation of legumes in the diets of school-going children could play a vital role in eradicating protein-energy malnutrition (PEM), especially in developing countries and in developing Afro-Asian countries.

## Author contributions

NS conceived and edited the final manuscript. PJ contributed to initial draft writing and editing. MU contributed to initial draft, tables, and figures. SL contributed to initial draft writing and editing. All authors contributed to the writing and editing, and approved the manuscript.
